# Association between gambling disorder and suicide mortality: a comparative cohort study using Norwegian health registry data

**DOI:** 10.1016/j.lanepe.2024.101127

**Published:** 2024-11-11

**Authors:** Joakim Hellumbråten Kristensen, Carl Michael Baravelli, Tony Leino, Ståle Pallesen, Mark D. Griffiths, Eilin Kristine Erevik

**Affiliations:** a Department of Psychosocial Science, Faculty of Psychology, University of Bergen, Post Box 7807, Bergen 5020, Norway; b Norwegian Competence Center for Gambling and Gaming Research, University of Bergen, Post Box 7807, Bergen 5020, Norway; c Department of Disease Burden, Norwegian Institute of Public Health, Post Box 973 Sentrum, Bergen 5808, Norway; d International Gaming Research Unit, Psychology Department, Nottingham Trent University, 50 Shakespeare Street, Nottingham NG1 4FQ, United Kingdom

**Keywords:** Pathological gambling, Suicidality, Addiction, Register, Standardized mortality ratios

## Abstract

**Background:**

Gambling disorder has been consistently linked to suicidal ideation and suicide attempts, but few studies have investigated the association between gambling disorder and suicide mortality. This study examined the risk of suicide mortality associated with gambling disorder compared to the general population and other patient groups.

**Methods:**

A registry-based cohort study was conducted employing individual-level linked and aggregated data from Norwegian nationwide health registries. The study population comprised all patients with gambling disorder in Norway (*n* = 6899) for the period 2008 to 2021. Standardized mortality ratios were estimated to assess suicide risk among gambling disorder patients against the adult Norwegian general population. Cox regressions were used to estimate hazard ratios comparing suicide risk among patients with gambling disorder to 12 comparison groups comprising patients treated for other conditions (*n* = 391,897).

**Findings:**

Suicide was the leading cause of death among patients with gambling disorder (37 of 148 deaths; 25%). Patients with gambling disorder had a higher suicide risk than the general population (standardized mortality ratio = 5.12, 95% CI [3.71; 7.06]), and 5 of 12 patient groups with other conditions. Suicide risk was not significantly different when compared to that of patients with anxiety disorders, personality disorders, or depression. However, suicide risk was lower among patients with gambling disorder than patients with substance use disorders, alcohol dependence, psychotic disorders, or mood disorders.

**Interpretation:**

Norwegian patients with gambling disorder have an elevated risk of suicide mortality but the risk is similar to or lower than other patient groups known to be at increased suicide risk.

**Funding:**

*Norwegian Competence Center for Gambling and Gaming Research* and the Faculty of Psychology at the 10.13039/501100005036University of Bergen (no specific grant).

**Research in context**

**Evidence before this study**

In January 2024, we published a comprehensive meta-analytic literature review of the available evidence on the relationship between gambling disorder/problem gambling and suicidality (i.e., suicidal ideation, suicide attempts, and suicide mortality) including a total of 107 primary studies. For the present study, two additional literature searches were conducted in Medline and PsycINFO on June 6, 2024, in order to identify reviews on the topic as well as recently published primary studies. Three additional primary studies and four reviews were found. The complete search strategy and inclusion criteria for both the present and original literature searches can be found in Appendix A. There is substantial evidence that gambling disorder and problem gambling are associated with an increased risk of suicidal ideation and suicide attempts. However, there are very few studies investigating the association between gambling disorder and suicide mortality. Only two relevant cohort studies have previously been published. No previous study has investigated whether individuals with gambling disorder have a higher or lower risk of suicide mortality compared to other relevant risk/disordered groups.

**Added value of this study**

The present study extends previous research by being (to date) the largest cohort study examining the association between gambling disorder and suicide mortality. It is the very first study to compare the risk of suicide mortality among patients with gambling disorder with other patient groups. Gambling disorder was associated with a significantly increased risk of suicide mortality compared to the general population. The risk of suicide mortality was higher for patients with gambling disorder than among patients suffering from a random mental disorder, a random somatic disorder, behavioral syndromes associated with physiological disturbances and physical factors, developmental disorders, and behavioral and emotional disorders with onset usually occurring in childhood and adolescence. However, the risk was similar (anxiety disorders, personality disorders, depression) or lower compared to other patient groups (psychotic disorders, mood disorders, alcohol dependence, substance use disorders) known to have a high risk of suicide mortality.

**Implications of all the available evidence**

The current evidence attests to the fact that gambling disorder is a severe psychiatric condition that warrants clinical, political, and public attention and intervention. Patients with gambling problems should routinely be screened for suicidality. Although causal evidence is lacking and needs to be investigated in future studies, the current literature suggests that efforts to prevent gambling disorder and subsyndromal gambling problems may contribute to preventing suicides.

## Introduction

A minority of individuals participating in gambling lose control and develop gambling disorder (GD). In the 11th revision of the *International Classification of Diseases* (ICD-11),^1^ GD is recognized as a behavioral addiction, characterized as a pattern of persistent or recurrent maladaptive gambling behavior that is manifested by impaired control over gambling, elevated priority on gambling, and continuation or escalation of gambling despite the occurrence of negative consequences. The GD diagnosis is usually assigned if the maladaptive gambling behavior is evident over at least 12 months and results in significant distress and/or impairment in normal functioning to the individual.^1^ GD is also recognized as a behavioral addiction in the fifth edition of the *Diagnostic and Statistical Manual of Mental Disorders* (DSM-5),^2^ in which GD is considered present when meeting at least 4 of 9 the addiction-related criteria specified in the manual within a 12-month period.^2^ Most scientific studies on GD are based on GD conceptualizations from the different editions of DSM, while the ICD-classification system is the main framework used in clinical practice and international recording outside the US.^3^^,^^4^

The prevalence of GD is approximately 0.4% to 0.6% of the general adult population worldwide.^4^ However, a larger proportion, 1.4% of adults globally,^5^ experience subsyndromal GD symptoms, commonly referred to as ‘problem gambling’.^4^ In Norway, where the present study was conducted, the most recent population study, conducted in 2022, estimated the prevalence of problem gambling among adults aged 16 to 74 to be 0.6%, corresponding to approximately 23,000 individuals.^6^ However, the number of individuals seeking formal treatment for GD provided by the Norwegian specialist healthcare service is low compared to the prevalence of problem gambling. From 2008 to 2021 the median annual number of patients receiving formal treatment for GD was 978, ranging from 349 patients in 2008 to 1736 in 2019.^7^

GD is associated with severe adverse effects and distress for both the individual and their close ones; including financial strain, relationship breakdowns, job loss, significant emotional distress, legal problems, and decrements in somatic and mental health^4^^,^^8^; which may put individuals with GD at an increased risk for suicide. Recent meta-analyses have confirmed a consistent and significant relationship between problem gambling/GD and suicidality.^9^ However, most investigations on the topic have examined suicidal ideation and non-fatal suicide attempts, and only very few studies have investigated the association between GD and suicide mortality.^9^ Moreover, most existing research on the relationship between problem gambling/GD and suicide mortality has been based on studies examining different risk factors among individuals who died by gambling-related suicide, ecological studies, or qualitative research designs,^10^^,^^11^ which do not allow for investigating the incidence- or the risk of suicide mortality associated with GD.

To date, only two published studies have assessed the risk of suicide mortality associated with GD using population-level cohort designs. Using a health-registry approach, Karlsson and Håkansson^12^ reported that suicide was the leading cause of death (31%; 21 of 67 deaths) within the Swedish cohort of patients treated for GD (F63.0) between 2005 and 2016 (*n* = 2099). Moreover, Swedish patients with GD had a 15-fold higher risk of suicide mortality compared to the general population.^12^ Similarly, Pavarin et al. reported that patients with GD (F63.0) in the Emilia-Romagna region of Italy between 1992 and 2018 (*n* = 826) had an increased suicide mortality risk, although the increased risk was not statistically significant.^13^

Because the two previous cohort studies only compared the risk of suicide mortality to the general population, it is currently unclear whether patients with GD are at a different suicide risk compared to other specific risk groups for suicide. Several mental health disorders other than GD are associated with an elevated risk of suicide mortality compared to the general population.^14^ However, comparing the risk differences between different mental health disorders across studies is difficult because of methodological differences between studies. Therefore, research directly investigating the association between GD and suicide mortality in relation to other known risk groups for suicide is warranted to evaluate the suicide risk associated with GD with more scrutiny.

Against this backdrop, the present study aimed to expand on previous studies by (i) assessing the risk of suicide mortality among Norwegian residents with GD; and (ii) comparing the risk of suicide mortality among patients with GD against other patient groups. Based on previous findings,^12^^,^^13^ it was expected that Norwegian individuals with GD would have an increased risk of suicide mortality compared to the Norwegian general population. No hypotheses were made for comparisons with other patient groups because these analyses were exploratory.

## Methods

### Study design and data sources

A cohort study was conducted by utilizing aggregated population-level data and individual-level data from nationwide Norwegian population-based sources: The Norwegian Patient Registry (NPR)^7^ and the Norwegian Cause of Death Registry (CDR).^15^ The study period ranged from January 1, 2008, to December 31, 2021. The NPR was used to identify the study population (i.e., patients with GD) and 12 patient comparison groups. The NPR is a nationwide health registry that holds administrative data from public and private contract (inpatient and outpatient) specialist healthcare services in Norway which requires a referral from primary healthcare providers for access.^16^ Since 2008, the NPR has recorded individual-level patient data based on diagnostic codes according to the ICD-10,^17^ which are linked to patients’ unique national personal identification number assigned to all residents of Norway. For the GD group, the data retrieved from the NPR included patients’ sex, birth year, and all mental- and somatic health conditions by year according to the ICD-10 classifications. Only patients’ sex, birth year, diagnosis- and year for inclusion were obtained for the patient comparison groups.

The primary outcome was suicide mortality, defined as dying during the study period with intentional self-harm (X60-X84 or Y87) registered as the underlying cause of death. Information about the outcome was obtained through the CDR. The CDR is a nationwide health registry that has almost full coverage (98%) of all deaths of Norwegian residents, including Norwegian residents dying abroad.^18^ The registered underlying cause of death is based on the death certificate by the attending physician.^18^ For the Norwegian general population aged 20–89 years, aggregated suicide mortality rates were obtained from the CDR in terms of the number of suicide deaths observed in the study period stratified by the year of death, sex, and 5-year age groups and divided by the mid-year population of the respective strata. For all patients identified through the NPR (i.e., patients with GD and patient comparison groups), information on mortality status and the year- and cause of death according to ICD-10 classifications was obtained by an individual-level record linkage of CDR data to the NPR data. The record linkage was performed in May 2023 utilizing patients’ unique national identification number which allows for individual-level record linkage across nationwide population-based registries in Norway.

The present study was approved by the Regional Committee for Medical Research Ethics in Northern Norway (ref. 458447) which exempted the study from obtaining informed consent in accordance with Norwegian legislation.

### Study population and comparison groups

Two distinct source populations were used for the present study; the entire Norwegian population alive in the study period (2008–2021) for comparing the risk of suicide mortality among patients with GD to the general population; and all patients registered in the NPR during the study period for comparing patients with GD to other patient groups.

The study population comprised 7016 patients who were registered in the NPR to have received a diagnosis of GD (pathological gambling, F63.0 according to the ICD-10) during the study period by the specialist healthcare service in Norway. Patients with GD who were younger than 18 years old when receiving their first GD diagnosis were excluded from the dataset (*n* = 117) to mitigate potential classification bias, considering that the legal gambling age in Norway is 18 years old and that a GD diagnosis among patients below the legal gambling age might reflect video game problems as some clinicians regard GD as the closest approximation in the absence of formal recognition of ‘gaming disorder’ in the ICD-10. Results from sensitivity analyses reproducing the main results when also including patients with GD who received their GD diagnosis before 18 years of age are available in Supplementary Tables S1 and S2 in Appendix B. The sensitivity analyses did not yield results statistically significantly different from the original estimates.

The comparison groups comprised 12 separate patient groups of individuals who in the study period had received a mental/behavioral disorder (or somatic) diagnosis by the specialist healthcare service. The first two comparison groups comprised broad groups of patients with a random mental/behavioral disorder (F10–F99) and a group of patients diagnosed with a random somatic condition (ICD-10 Chapters A, B, C, D, E, G, H, I, J, K, L, M, N, P, and Q). The remaining comparison groups comprised diagnostic categories according to the broad categories of Chapter V in the ICD-10 and included patients with: substance use disorders (F10–F19), psychotic disorders (F20–F29), mood disorders (F30–F39), anxiety disorders (F40–F48), behavioral syndromes associated with physiological disturbances and physical factors (F50–F59), personality disorders (F60–F69), developmental disorders (F80–F89), and behavioral and emotional disorders with onset usually occurring in childhood and adolescence (F90–F98). Additionally, depression (F32–F33) and alcohol dependency (F10.2) were included as comparison groups specifically because these conditions have been particularly associated with increased risk of suicide mortality.^14^

The individuals within the comparison groups were drawn randomly from the NPR but had to be born before 2004. Patients in the comparison groups could have received multiple diagnoses but could not have GD. The comparison groups were drawn to be six times larger than the GD group (all patients with the specific diagnoses/diagnosis were included when not a sufficient number of patients had received the specific diagnoses/diagnosis). This resulted in comparison groups with sample sizes ranging from *n* = 34,032 to *n* = 42,096, and a total of 391,897 patients were included in comparison groups. The decision to draw comparison groups six times larger than the GD group was informed by a provisional and an a priori power analysis using the *power.smr.test* module in R.^19^ The research team originally planned to compare suicide risk among patients with GD to the patient comparison groups by estimating standardized mortality ratios (SMRs). However, the analytic approach for these comparisons was changed post-data collection from SMR analyses to Cox proportional hazards regression models as outlined in the ‘Data Analysis’-section of this article. The SMR power analysis, assuming a GD population of 40,000 person-years and an annual crude suicide mortality rate of 0.00012 of the Norwegian general population, demonstrated that including comparison groups of equal size as the expected GD population would result in low statistical power (44%) for detection a practically relevant significant effect (i.e., SMR = 2.0)^20^ at the .05 significance level. Increasing the number of person-years for the comparison group(s) to six times that of the expected GD population (i.e., 240,000 person-years) yielded satisfactory statistical power at approximately 99%. All patients were followed from the index episode (year of received diagnosis) until death or study end (December 31, 2021), during which patients were censored from the study if they died from causes other than suicide.

### Data Analysis

Descriptive analyses were conducted to assess the characteristics of patients with GD in terms of sex, age, and comorbidity. The descriptive analyses were stratified on suicide mortality and significance tested using two-sided independent sample *t*-tests and chi-squared tests to identify potential effect modifiers of suicide mortality. Crude mortality rates of suicide mortality per 1000 person-years were estimated for all patient groups by dividing the number of suicide deaths by the number of person-years at risk within the group and multiplying by 1000.

Standardized mortality ratios (SMRs) were estimated to assess the risk of suicide mortality associated with GD compared to the Norwegian general population. The SMR is a mortality rate ratio which in the present study reflected the number of observed suicide deaths in the GD population divided by the expected number of deaths given that the suicide mortality rate would be equal to the general population. The expected number of suicide deaths was estimated by multiplying patients’ specific person-years at risk by the corresponding, sex-, age- (5-year age groups), and calendar-year specific (2008–2021) suicide mortality rates in the general population. The *Lexis* method^21^ was used to assign the specific person-years at risk to the respective suicide mortality rates in each stratum and calendar year for patients transitioning between age bands during follow-up. The SMR and corresponding 95% confidence intervals (CIs) were then modeled by Poisson regression models with the number of observed suicide deaths as the dependent variable and the log expected suicide deaths as the offset.^21^^,^^22^ SMR analyses were conducted for age categories 20–89, 20–49, and 50–89 years, and for men and women separately. Additionally, psychiatric comorbidity was assessed as a potential effect modifier on the relationship between GD and the risk of suicide by stratifying SMR analyses among patients with GD aged 20–89 on the presence of having one or more F-diagnosis (F00–F99).

A series of Cox proportional hazards regression models were estimated to assess the hazard ratio (HR) of suicide mortality between the GD group and the patient comparison groups. Given the large number of comparisons of patient groups, the research team prioritized selecting the lowest possible number of model variations while maintaining robustness and applicability across the most possible comparisons. Therefore, two sets of Cox regressions were conducted for each comparison to account for violations of the proportionality assumption of the Cox model. The first set of Cox regressions was conducted using chronological age as the timescale, in which the entry time was age at the index episode and the exit time was age at the event (suicide mortality) or censoring (i.e., age at death by other cause than suicide, or study end). Suicide mortality was regressed on patient group and adjusted for age (as the timescale) and sex. The Cox regressions were stratified on 25-year interval birth cohorts to adjust for calendar effects related to both the exposure and outcome. Broad birth cohorts of 25 years were chosen because narrower intervals resulted in several small strata and few events within each stratum, consequently leading to violations of the proportionality assumption for several models.

The second set of Cox regression analyses was conducted using time-on-study as the timescale (i.e., years since the index episode) and regressing suicide mortality on patient group, adjusted for sex and age at the time of censoring as covariates. The entry time was year of the index episode, and the exit time was year of suicide mortality or censoring (i.e., age at death by other cause than suicide, or study end). Age was included as a covariate (or as the timescale) in both sets of Cox regression models because rates of GD, other mental/behavioral disorders, and suicide mortality change with age.^4^^,^^23^^,^^24^ Similarly, sex was included as a covariate to adjust for sex differences in the rates of the exposure and outcome variable, particularly because men are overrepresented in the GD population and suicide deaths compared to women.^4^^,^^23^

The proportionality assumption of the Cox regression models was examined by visual inspection of Schoenfeld residuals against the timescale. Medical information about the cause of death was missing for 481 death certificates of the total 25,568 patient deaths (1.9%; no causes of death were missing from the GD group). These deaths were coded as death due to other causes than suicide and patients were retained for the analysis. No other data from NPR or CDR had missing values. The descriptive analyses were conducted using IBM SPSS (version 28.0.1.1). The SMR and Cox regression analyses were conducted using R (version 4.3.0)^25^ with the *Epi* (version 2.47.1)^26^ and *Survival* (version 3.5–5)^27^ packages, respectively.

### The role of the funding source

The present study was fully funded by the *Norwegian Competence Center for Gambling and Gaming Research* and the Faculty of Psychology at the University of Bergen (no specific grant). The funding sources exerted no influence on the present study design, data collection, data analysis, data interpretation, or writing of the article. Data from the Norwegian Patient Registry and the Norwegian Cause of Death Registry have been used in the present study. The interpretation and reporting of these data are the sole responsibility of the authors, and no endorsement by the Norwegian Patient Registry or the Norwegian Cause of Death Registry is intended nor should be inferred.

## Results

### Descriptive analyses of patients with gambling disorder

A total of 6899 patients, 5651 men (81.9%) and 1248 women (18.1%) with GD were identified. Patients were between 18 and 67 years of age when receiving their first GD diagnosis (M = 36.8, SD = 11.3). The total number of person-years of patients with GD was 38,965 and the mean follow-up time was 5.6 years (SD = 3.8, range = 0.5–13). The clinical characteristics of patients with GD in terms of comorbid somatic health and mental health diagnoses are displayed in Table 1.

**Table 1:** Comorbid somatic health and mental health diagnoses in patients with gambling disorder (n = 6899) received between 2008 and 2021 by the Norwegian specialist health service, stratified on suicide mortality.

ICD-10 code	Disease	Total GD sample *n* (%)	Non-suicide *n* (%)	Suicide *n* (%)
AB	Certain infectious and parasitic diseases	994 (14.4%)	985 (14.4%)	9 (24.3%)^ns^
CD	Neoplasms, diseases of the blood, and diseases involving the immune mechanism	1422 (20.6%)	1416 (20.6%)	6 (16.2%)^ns^
E	Endocrine, nutritional, and metabolic diseases	1300 (18.8%)	1287 (18.8%)	13 (35.1%)^a^
F00–F99	Mental and behavioral disorders	4069 (59.0%)	4039 (58.9%)	30 (81.1%)^b^
F00–F09	Organic, including symptomatic and mental disorders	117 (1.7%)	116 (1.7%)	1 (2.7%)^ns^
F10–F19	Mental and behavioral disorders due to psychoactive substance use	1700 (24.6%)	1681 (24.5%)	19 (51.4%)^c^
F10	Mental and behavioral disorders due to use of alcohol	1099 (15.9%)	1089 (15.9%)	10 (27.0%)^ns^
F11–F19	Mental and behavioral disorders due to use of drugs	1096 (15.9%)	1081 (15.8%)	15 (40.5%)^c^
F20–F29	Schizophrenia, schizotypal, and delusional disorders (psychotic disorders)	285 (4.1%)	280 (4.1%)	5 (13.5%)^b^
F30–F31	Bipolar disorders	350 (5.1%)	346 (5%)	4 (10.8%)^ns^
F32–F33	Depressive disorders	1996 (28.9%)	1973 (28.8%)	23 (62.2%)^c^
F34–F39	Other mood disorders	253 (3.7%)	250 (3.6%)	3 (8.1%)^ns^
F40–F48	Neurotic, stress-related, and somatoform disorders (anxiety disorders)	2229 (32.3%)	2209 (32.3%)	20 (54.1%)^b^
F50	Eating disorders	121 (1.8%)	121 (1.8%)	0 (0.0%)^ns^
F51	Nonorganic sleep disorders	64 (0.9%)	63 (0.9%)	1 (2.7%)^ns^
F52	Sexual disorders	24 (0.3%)	24 (0.3%)	0 (0.0%)^ns^
F60–F62	Personality disorders	793 (11.5%)	787 (11.5%)	6 (16.2)^ns^
F63	Impulse control disorders (excluding F63.0)	193 (2.8%)	193 (2.8%)	0 (0.0%)^ns^
F64–F69	Other disorders of personality	32 (0.5%)	32 (0.5%)	0 (0.0%)^ns^
F70–F79	Mental retardation	54 (0.8%)	54 (0.8%)	0 (0.0)^ns^
F80–F89	Pervasive and specific developmental disorders	179 (2.6%)	177 (2.6%)	2 (5.4%)^ns^
F90–F98	Behavioral and emotional disorders with onset usually occurring in childhood and adolescence	712 (10.3%)	708 (10.8%)	4 (10.8%)^ns^
F99	Unspecified disorder	463 (6.7%)	457 (6.7%)	6 (16.2%)^a^
G	Diseases of the nervous system	1678 (24.3%)	1663 (24.2%)	15 (40.5%)^a^
H	Diseases of the eye and the ear	1846 (26.8%)	1841 (26.8%)	5 (13.5%)^ns^
I	Diseases of the circulatory system	1387 (20.1%)	1371 (20.0%)	16 (43.2%)^c^
J	Diseases of the respiratory system	1818 (26.4%)	1804 (26.3%)	14 (37.8%)^ns^
K	Diseases of the digestive system	2399 (34.8%)	2386 (34.8%)	13 (35.1)^ns^
L	Diseases of the skin	1682 (24.4%)	1672 (24.4%)	10 (27.0%)^ns^
M	Diseases of the musculoskeletal system	2877 (41.7%)	2864 (41.7%)	13 (35.1%)^ns^
N	Diseases of the genitourinary system	1922 (27.9%)	1911 (27.8%)	11 (29.7%)^ns^
O	Pregnancy, childbirth, and the puerperium	429 (6.2%)	429 (6.3%)	0 (0.0%)^ns^
Q	Congenital malformations	293 (4.2%)	293 (4.3%)	0 (0.0%)^ns^
R	Symptoms, signs, and findings, not elsewhere classified	4100 (59.4%)	4077 (59.4%)	23 (62.2%)^ns^
ST	Injury, poisoning, and other consequences of external causes	4016 (58.2%)	3992 (58.2%)	24 (64.9%)^ns^
V01–X59	Accidents	610 (8.8%)	607 (8.8%)	3 (8.1%)^ns^
X60–X84	Intentional self-harm	258 (3.7%)	250 (3.6%)	8 (21.6%)^c^
X85–Y09	Assault	52 (0.8%)	52 (0.8%)	0 (0.0%)^ns^
Y10–Y34	Events of undetermined intent	8 (0.1%)	7 (0.1%)	1 (2.7%)^a^
Y40–Y98	Other external causes	214 (3.1%)	211 (3.1%)	3 (8.1%)^ns^

*Note.* Categorized according to the *International Classification of Disease* 10th *revision* (ICD-10); GD = gambling disorder; Two-sided chi-squared test: ns = no statistically significant difference.

a *p* < 0.5.

b *p* < 0.01.

c *p* < 0.001.

During the study period, 148 patients with GD (114 men and 34 women) died. Suicide was the leading cause of death (25.0%, *n* = 37) followed by neoplasms (C00-D48; 24.3%, *n* = 36), diseases of the circulatory system (I00–I99; 14.2%, *n* = 21), and accidental poisoning (X40-X49; 12.8%, *n* = 19). Among the patients with GD who died by suicide, 32 were men and five were women. The mean age at study exit (i.e., suicide mortality or censoring) was similar for those who died by suicide (M = 43.1; SD = 13.0) and those who did not (M = 42.4; SD = 11.9; *p* = 0.71). Table 1 further shows that patients with GD who died by suicide had a significantly higher prevalence of mental- and behavioral diagnoses, diseases of the nervous system, diseases of the circulatory system, and recorded contact with specialist healthcare due to intentional self-harm, compared to patients with GD who did not die by suicide.

### Risk of suicide mortality compared to the general population

The results comparing suicide mortality risk for patients with GD to the general population are presented in Table 2. Patients with GD aged 20–89 years were found to have a higher risk of suicide mortality than the general population, SMR = 5.12 (95% CI [3.71; 7.06]). Higher SMRs were found for patients with GD aged 50–89 years and for female patients with GD (see Table 2), but the SMRs were not statistically different across strata as evidenced by overlapping CIs. The SMR analysis stratified on psychiatric comorbidity yielded SMR = 6.93 (95% CI [4.85; 9.91]), versus SMR = 2.41 (95% CI [1.15; 5.06]) when including only patients with GD without psychiatric comorbidity. However, the effect modification was not statistically significant.

**Table 2:** Standardized mortality ratios of suicide mortality among patients with gambling disorder against the general population.

Sex	Age group	Person-years	Observed suicide mortality	CMR per 1000 person-years	SMR (95% CI)
Men and women	Age 20–89	38748.5	37	0.955	5.12 (3.71; 7.06)
	Age 20–49	30372.5	27	0.889	4.75 (3.26; 6.93)
	Age 50–89	8376.0	10	1.194	6.46 (3.48; 12.01)
Men	Age 20–89	31778.5	32	1.007	4.88 (3.45; 6.90)
Women	Age 20–89	7186.5	5	0.696	7.43 (3.09; 17.84)

CMR = crude mortality rate; SMR = standardized mortality ratio; CI = confidence intervals.

### Risk of suicide mortality compared to other patient groups

The results from Cox regression analyses comparing patients with GD to other patient groups on suicide mortality risk are presented in Table 3. Patients with GD had a (marginally) statistically significantly higher suicide risk than patients with a random mental/behavioral disorder and patients with a random somatic condition. Moreover, patients with GD had a higher suicide risk compared to patients with behavioral syndromes associated with physiological disturbances and physical factors, developmental disorders, and patients with behavioral and emotional disorders with onset usually occurring in childhood and adolescence (see Table 3). Patients with GD were found to have a lower suicide risk than patients with substance use disorders, alcohol dependence, psychotic disorders, and mood disorders. No statistically significant differences in risks were found when comparing patients with GD to patients with depression, anxiety disorders, and personality disorders in either set of Cox regression models (see Table 3). The two sets of Cox regression models based on different timescales yielded results with similar patterns across all analyses, although HRs became statistically significant in the models using time-on-study as the timescale when comparing patients with GD to patients with a random mental/behavioral disorder and patients with mood disorders (see Table 3). Kaplan–Meier survival curves were produced for each comparison for both timescales and are available in Appendix C.

**Table 3:** Cox regression models examining hazard ratios of suicide mortality among patients with gambling disorder against other patient groups.

Reference group (ICD-10 codes)	*n*	Person-years	Observed suicide mortality	Crude suicide mortality rate per 1000 person-years	Hazard ratio adjusted for age, sex, and calendar effects (95% CI)^a^	Hazard ratio adjusted for age at censoring and sex (95% CI)^b^
Random mental/behavioral disorder (F10–F99)	42,096	232,913	114	0.49	1.44 (0.97; 2.14)	1.57 (1.07; 2.31)^c^
Random somatic condition (Chapters A, B, C, D, E, G, H, I, J, K, L, M, N, P, Q)	42,095	231,970	26	0.11	–	7.04 (4.13; 12.00)^e^
Substance use disorders (F10–F19)	42,096	220,956	368	1.67	0.53 (0.38; 0.75)^e^	–
Alcohol dependence syndrome (F10.2)	38,284	207,477	268	1.29	0.62 (0.43; 0.88)^d^	–
Psychotic disorders (F20–F29)	34,032	198,867	396	1.99	–	0.39 (0.28; 0.56)^e^
Mood disorders (F30–F39)	42,093	229,457	240	1.04	0.73 (0.51; 1.05)	0.66 (0.46; 0.93)^c^
Depression (F32–F33)	42,096	230,940	163	0.71	1.02 (0.70; 1.48)	–
Anxiety disorders (F40–F48)	42,095	234,421	115	0.49	1.35 (0.91; 2.0)	1.91 (0.89; 1.93)
Behavioral syndromes associated with physiological disturbances and physical factors (F50–F59)	42,095	238,078	87	0.37	1.84 (1.17; 2.90)^d^	1.93 (1.24; 3.01)^d^
Personality disorders (F60–F69)	42,095	237,617	197	0.83	0.94 (0.65; 1.35)	0.93 (0.65; 1.33)
Developmental disorders (F80–F89)	34,825	222,508	59	0.27	2.18 (1.31; 3.61)^d^	3.76 (2.26; 6.23)^d^
Behavioral and emotional disorders with onset usually occurring in childhood and adolescence (F90–F98)	42,096	240,833	67	0.28	2.17 (1.39; 3.39)^e^	3.23 (2.05; 5.09)^e^

- = Proportionality assumption for the Cox regression model was not met; CI = confidence intervals.

a Cox regression model using chronological age as the timescale adjusted for sex and stratified on birth cohorts.

b Cox regression model using time-on-study as the timescale adjusted for age at censoring and sex.

c *p* < 0.05.

d *p* < 0.01.

e *p* < 0.001.

## Discussion

Suicide was the leading cause of death (25%) among Norwegian patients with GD who had a 5-fold increased risk of suicide mortality compared to the general population. Moreover, patients with GD had a higher suicide risk than 5 of 12 patient groups with other conditions, including any random patient treated for a mental/behavioral and/or somatic health condition in specialist healthcare domains. Still, the risk was similar to some patient groups known to have an increased risk of suicide (i.e., patients with anxiety disorders, personality disorders, and depression), and lower risk than patients with psychotic disorders, mood disorders, substance use disorders, and alcohol dependence syndrome.

The observed increased risk of suicide mortality associated with GD when compared to the general population is consistent with findings from the two previously published comparable studies.^12^^,^^13^ However, the present SMR estimate of 5.12 was significantly lower than the corresponding estimate of 15.1 found in Sweden.^12^ The discrepancy in the SMR estimates could be related to potential differences in diagnostic practices between the two countries. The number of patients with GD is higher in Norway than in Sweden, although Sweden has about twice the population size compared to Norway and is comparable in terms of health and welfare structure, culture, political system, and prevalence of problem gambling.^6^^,^^28^ Accordingly, it is possible that the GD diagnosis is less known or used by clinicians in Sweden and that the GD diagnosis is primarily given to patients with high symptom severity, potentially explaining the differences in the SMR estimates of suicide mortality found among the Swedish and Norwegian GD cohorts.

The present study is novel because it is the first to assess suicide mortality among patients with GD compared to multiple patient groups with other conditions. The observed differences in suicide risk may be related to characteristics inherent to the specific diagnoses (e.g., decreased inhibitions due to substance use). Yet, the risk differences may also be driven by comorbidity typically associated with a specific condition rather than the particular condition itself. Similarly, risk differences may be influenced by competing risks wherein certain patient groups might have a higher risk of mortality from other condition-specific causes than suicide, potentially precluding or reducing the likelihood of suicide mortality for these patients.^29^ The observed risk differences in suicide mortality may also be driven by specific help-seeking behaviors which may vary across conditions due to, for example, differences in mental health literacy, fear of stigma, shame related to specific conditions, prognosis, and perceived or factual availability of condition-specific treatment opportunities.^30^, ^31^, ^32^ Regarding the latter, formal treatment of GD in Norway is provided by the specialist health service and is available for patients within all health regions in Norway. The economic burden for patients related to formal treatment is also relatively low due to a cost-sharing ceiling (approximately 290 € in 2022) in which patients are exempted from copayments for all public healthcare services for the rest of the calendar year when reaching the ceiling. Still, the actual accessibility of GD-specific treatment may be more limited. A survey among all addiction specialist treatment facilities in Norway indicated that only 37% specifically provided treatment for GD.^33^ Moreover, several treatment facilities report having limited clinical experience and routines regarding GD treatment compared to other conditions.^33^ Accordingly, variations in actual availability and clinical competence about GD could affect treatment outcomes and, consequently, potentially influence suicide risk. Nevertheless, when comparing patients with GD against other patient groups the results suggest that the increased suicide risk does not appear to be exclusive to patients with GD. Therefore, the present results can be interpreted to indicate that the observed relationship between GD and suicide mortality may be driven by a common underlying psychopathological “g-factor” of increased suicide risk associated with mental health disorders in general, and not unique characteristics of GD, which is a notion consistent with some previous research.^34^

A major constraint of the present study is its limitation in establishing a causal relationship between GD and suicide mortality. High levels of comorbidity were found within the GD cohort in which several of these conditions themselves are associated with increased suicide risk,^14^^,^^35^ and the present study design prevents making inferences on directionality or the underlying processes between GD, comorbidity, and suicide mortality. Moreover, the present dataset did not include information on social variables, potentially traumatic life events, or health conditions not diagnosed within the specialist health service, which all may be important third factors related to GD and suicide mortality. Additionally, the dataset did not contain data on comorbid mental health diagnoses in the patient comparison groups which may explain differences in suicide mortality across groups.

Another limitation is the highly likely influence of a selection effect (e.g., being diagnosed in specialist healthcare facilities) which hampers the generalizability of results and may bias the estimates. However, the direction of potential bias is unclear. The present study population comprises patients with GD who sought and were referred to specialized treatment, who are likely to have a higher symptom severity compared to individuals with GD who do not seek treatment.^36^ Therefore, the effects observed in the present study may be somewhat inflated. Still, most individuals with problem gambling/GD do not seek treatment.^36^ In Norway, an annual median of only 978 patients sought formal treatment for GD between 2008 and 2021^7^ while approximately 23,000 Norwegian adults were estimated to be experiencing problem gambling in 2022.^6^ Consequently, it is possible that some of the individuals with undiagnosed and untreated GD are at greater suicide risk compared to those who receive treatment. In addition, the proportionality assumption of the Cox regression model was violated in some of the comparisons of patients with GD to other patient groups. This suggests that the observed risk differences (i.e., HRs) between groups are likely to be subject to time-varying covariates that were not included/investigated in the models. Future studies may thus benefit from investigating the influence of such time-varying covariates (e.g., age-dependent gambling restrictions or economic factors) to better understand the dynamic relationship between GD and suicide risk.

Furthermore, the data in the NPR and CDR rely on diagnostic reports from attending clinicians which might be prone to systematic biases in diagnostic coding and are also normally not subject to validity checks,^16^^,^^18^ potentially introducing classification errors to both the exposure(s) and outcome of the present study. For instance, data from patients with GD below the age of 18 years (the legal gambling age in Norway) were excluded from this study to reduce potential classification bias as it is known that some clinicians use GD as a proxy diagnosis for (video) gaming disorder. However, this diagnostic coding-/classification bias may also be present in a subset of patients with GD older than 18 years who were included in the study. Moreover, accidental poisoning (X40-X49) was the fourth most frequent cause of death (12.8%) among patients with GD. However, this diagnostic category has been criticized for its lack of specificity, of which some deaths are likely to reflect misclassified suicides.^37^

Finally, it should be noted that although the GD nomenclature is used throughout this article, the current analysis and data are based on the F63.0 ‘pathological gambling’ diagnosis and the related criteria which have now been superseded. While there are some conceptual differences between the ICD-10 and the more recent DSM-5 and ICD-11 classifications, this has most likely a limited practical impact on the identification of patients seeking formal treatment for gambling-related problems in Norway. In practice, Norwegian clinicians use a wide range of diagnostic tools based on various versions of the DSM and ICD diagnostic manuals to identify gambling-related behavior and problems in need of treatment.^33^ However, the F63.0 diagnosis is the formal diagnosis assigned to these patients because the ICD-10 is the official coding system used in Norway’s specialist health service and has been used throughout the whole period from which the data stems.

In summary, the present study attests to previous studies showing that suicide is the most frequent cause of death among patients with GD and that GD is associated with an increased risk of suicide mortality when compared to the general population. The risk of suicide mortality is of a similar magnitude among patients with GD and other patient groups known to be at elevated suicide risk. Future studies are needed to investigate the role of comorbidity and various social factors and to elucidate relevant causal mechanisms in the relationship between GD and suicide mortality. Nonetheless, the present study’s findings underscore the importance of recognizing GD and problem gambling as serious mental health and public health concerns that warrant inclusion in suicide prevention strategies.

## Contributors

JHK, TL, SP, and EKE conceived and designed the study. JHK administrated the project under the supervision of EKE, SP, and TL. JHK conducted the analyses and drafted the initial manuscript. CMB provided methodological and statistical advice. TL accessed and verified the data, analyses, and results. All authors supported the interpretation of the findings and significantly contributed to editing the manuscript. All authors had final responsibility for the decision to submit the manuscript for publication.

## Data sharing statement

The datasets based on individual-linked data from the Norwegian Patient Registry and the Norwegian Cause of Death Registry presented in this article are not readily available due to Norwegian privacy regulations. Access to Norwegian individual-level health registry data is provided exclusively by approval from the Norwegian Regional Committees for Medical Research Ethics (https://www.forskningsetikk.no/en/about-us/our-committees-and-commission/rek/). Aggregated suicide mortality rates in the Norwegian general population are openly available from the Norwegian Institute of Public Health (https://norgeshelsa.no/norgeshelsa/) or by request to the Norwegian Cause of Death Registry (https://helsedata.no/en/).

## Declaration of interests

MDG has received research funding from *Norsk Tipping* (the gambling operator owned by the Norwegian government). MDG has received funding for a number of research projects in the area of gambling education for young people, social responsibility in gambling and gambling treatment from *Gamble Aware* (formerly the *Responsibility in Gambling Trust*), a charitable body that funds its research program based on donations from the gambling industry. MDG undertakes consultancy for various gambling companies in the area of player protection and social responsibility in gambling. The authors declare that the present research was conducted in the absence of any commercial or financial relationships that could be construed as potential conflicts of interest.
