# Supplementary material for: Association between gambling disorder and suicide mortality: a comparative cohort study using Norwegian health registry data
Source: Lancet Reg Health Eur. 2024 Nov 11;48:101127. doi: 10.1016/j.lanepe.2024.101127 (PMC11600009; doi:10.1016/j.lanepe.2024.101127)
Supplement: Appendix A–D [file mmc1.docx]

**Appendices: The Association Between Gambling Disorder and Suicide Mortality: A Comparative Cohort Study Using Norwegian Health Registry Data**

Appendix A Search Strategy and Inclusion Criteria Used in the Literature Review for ‘Research in Context’

Appendix B: Sensitivity Analyses

Appendix C: Kaplan-Meier Survival Plots

Appendix D: STROBE Checklist

**Appendix A: Search Strategy and Inclusion Criteria Used in the Literature Review for ‘Research in Context’**

Original literature search strategy in Kristensen et al., 2024 (All fields in Web of Science Core Collection, PsychINFO, PsycNet, Medline, CINAHL, ProQuest, Embase, Google Scholar, and reference lists of included studies, completed April 1, 2023, updated June 6, 2024):

("problem* gambl*" OR "gambling problem*" OR "gambling addiction" OR "pathological gambl*" OR "compulsive gambl* OR "gambling disorder" OR "disordered gambl*" OR ludomania) AND (suic* OR self-inflicted OR self-harm OR parasuicide)

Inclusion criteria for the original review:

(a) Addressed gambling problems specifically

(b) Addressed suicidal ideation, suicide attempts, and/or suicide mortality specifically

(c) Were reported in any European language

(d) Reported original empirical data on prevalence rates, or comparisons against individuals without gambling problems, on suicidal ideation, suicide attempts, and/or suicide among individuals with gambling problems (or sufficient data to calculate prevalence rates/effect sizes)

Exclusion criteria for the original review:

(a) reported findings on unproblematic/normal gambling behavior or behaviors that may cover gambling problems but are not limited to gambling

(b) reported findings concerning self-harm without lethal intent (i.e., non-suicidal self-injury)

(c) reported current risk/threat of suicide rather than suicidal ideation, suicide attempts, or suicide specifically

(d) case studies, qualitative studies, literature reviews/meta-analyses, and animal studies

Reference to original review:

Kristensen JH, Pallesen S, Bauer J, Leino T, Griffiths MD, Erevik EK. Suicidality among individuals with gambling problems: A meta-analytic literature review. *Psychol Bull* 2024;**150**(1):82-106.

Additional literature search to include review articles (Titles and abstracts in Medline and PsycINFO, completed June 6, 2024):

("problem* gambl*" OR "gambling problem*" OR "gambling addiction" OR "pathological gambl*" OR "compulsive gambl* OR "gambling disorder" OR "disordered gambl*" OR ludomania) AND (suic* OR self-inflicted OR self-harm OR parasuicide) AND (review OR meta-analysis))

Inclusion criteria for additional litterature search of reivew articles:

(a) Article was a qualitative review, systematic review, or meta-analysis

(b) Addressed gambling problems/gambling disorder specifically

(c) Addressed suicidal ideation, suicide attempts, and/or suicide mortality specifically

(d) Were reported in any European language

Exclusion criteria for additional litterature search of reivew articles:

(a) Primary studies and animal studies

**Appendix B: Sensitivity Analyses**

**Supplementary Table 1**

*Sensitivity standardized mortality ratio analyses of suicide mortality among patients with gambling disorder against the general population,* *including patients with gambling disorder diagnosed before the age of 18.*

| **Sex** | **Age group** | **Person-years** | **Observed suicide mortality** | **CMR per 1000 person-years** | **SMR (95% CI)** |
| --- | --- | --- | --- | --- | --- |
| Men and women | Age 20-89 | 39,903·0 | 37 | 0·927 | 5·05 (3·66; 6·97) |
|  | Age 20-49 | 31,527·0 | 27 | 0·856 | 4·67 (3·20; 6·81) |
|  | Age 50-89 | 8,376·0 | 10 | 1·194 | 6·46 (3·48; 12·01) |
| Men | Age 20-89 | 32,674·5 | 32 | 0·979 | 4·81 (3·40; 6·80) |
| Women | Age 20-89 | 7,228·5 | 5 | 0·692 | 7·40 (3·08; 17·79) |

*Note*. CMR = crude mortality rate; SMR = standardized mortality ratio; CI = confidence intervals.

**Supplementary Table 2**

*Sensitivity Cox regression models examining hazard ratios of suicide mortality among patients with gambling disorder against other patient groups, including patients with gambling disorder diagnosed before the age of 18.*

| **Reference comparison group**  **(ICD-10 codes)** | ***n*** | **Person-years** | **Observed suicide mortality** | **Crude suicide mortality rate per 1,000 person-years** | **Hazard ratio adjusted for age, sex, and calendar effects (95% CI)^1^** | **Hazard ratio adjusted for age at censoring and sex (95% CI)^2^** |
| --- | --- | --- | --- | --- | --- | --- |
| Random  mental/behavioral disorder (F10-F99) | 42,096 | 232,913 | 114 | 0·49 | 1·42 (0·96; 2·12) | 1·45 (1·05; 2·27)* |
| Random somatic condition (Chapters A, B, C, D, E, G, H, I, J, K, L, M, N, P, Q) | 42,095 | 231,970 | 26 | 0·11 | - | 6·85 (4·01; 11·7)*** |
| Substance use disorders (F10-F19) | 42,096 | 220,956 | 368 | 1·67 | 0·52 (0·37; 0·74)*** | - |
| Alcohol dependence syndrome  (F10.2) | 38,284 | 207,477 | 268 | 1·29 | - | - |
| Psychotic disorders (F20-F29) | 34,032 | 198,867 | 396 | 1·99 | - | 0·38 (0·27; 0·54)*** |
| Mood disorders (F30-F39) | 42,093 | 229,457 | 240 | 1·04 | 0·71 (0·49; 1·01) | 0·64 (0·45; 0·92)* |
| Depression (F32-F33) | 42,096 | 230,940 | 163 | 0·71 | 1·00 (0·68; 1·45) | - |
| Anxiety disorders (F40-F48) | 42,095 | 234,421 | 115 | 0·49 | 1·31 (0·89; 1·95) | 1·28 (0·87; 1·89) |
| Behavioral syndromes associated with physiological disturbances and physical factors (F50-F59) | 42,095 | 238,078 | 87 | 0·37 | 1·80 (1·14; 2·81)* | 1·88 (1·20; 2·93)** |
| Personality disorders (F60-F69) | 42,095 | 237,617 | 197 | 0·83 | 0·92 (0·64; 1·33) | 0·90 (0·62; 1·30) |
| Developmental disorders (F80-89) | 34,825 | 222,508 | 59 | 0·27 | 2·10 (1·28; 3·47)** | 3·58 (2·17; 5·90)*** |
| Behavioral and emotional  disorders with onset usually occurring in childhood and adolescence (F90-F98) | 42,096 | 240,833 | 67 | 0·28 | 2·11 (1·35; 3·30)*** | 3·11 (1·98; 4·90)*** |

*Note.* 1 = Cox regression model using chronological age as the timescale adjusted for sex and stratified on birth cohorts; 2 = Cox regression model using time-on-study as the timescale adjusted for age at censoring and sex; - = Proportionality assumption for the Cox regression model was not met; * = p < 0·05; ** = p < 0·01; *** = p < 0·001; CI = confidence intervals.

**Appendix C: Kaplan-Meier Survival Plots**

**Supplementary Figure 1**

Kaplan-Meier curves for comparing survival probability of suicide mortality among patients with gambling disorder compared to patients with random mental/behavioral disorder.


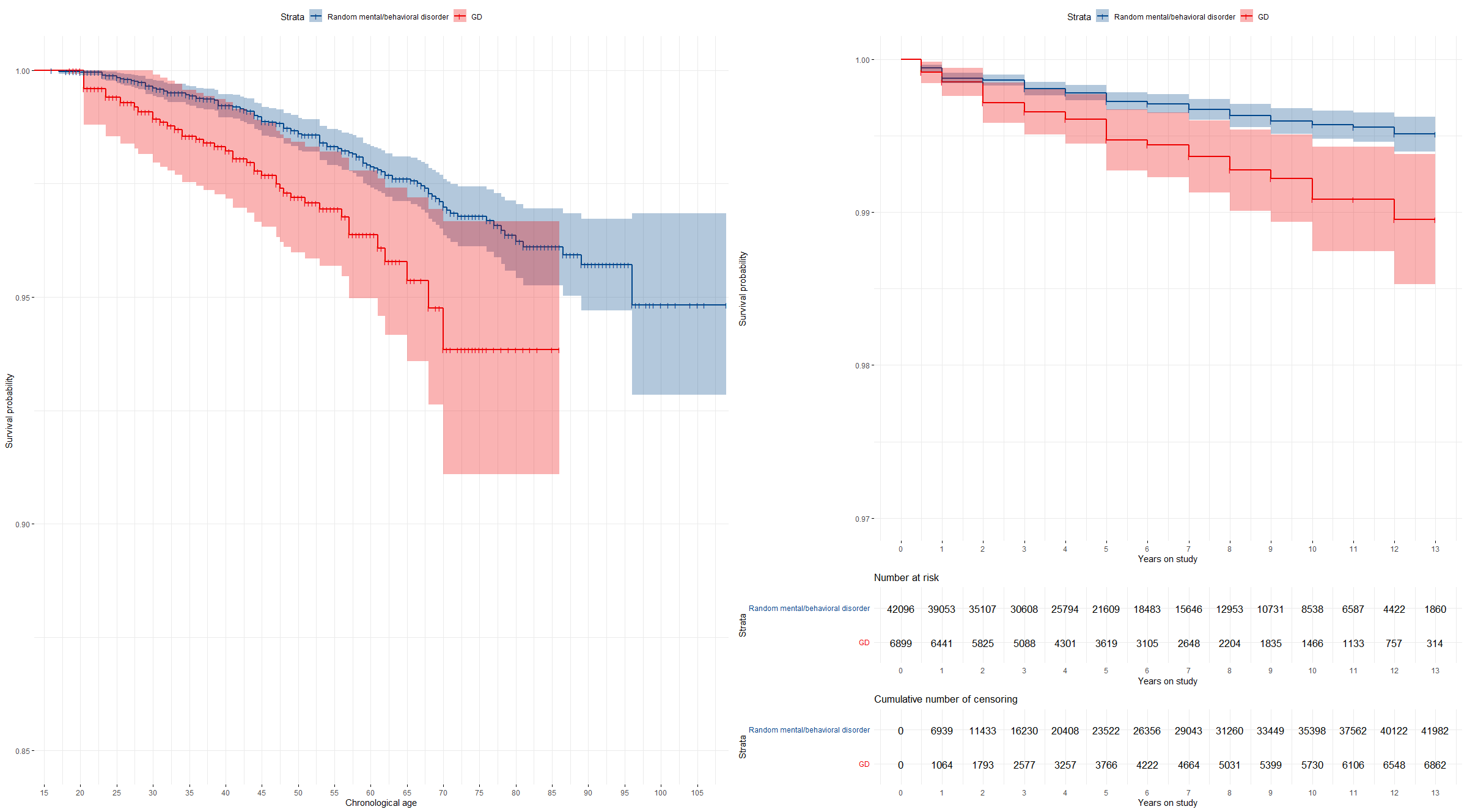


**Supplementary Figure 2**

Kaplan-Meier curves for comparing survival probability of suicide mortality among patients with gambling disorder compared to patients with random somatic condition.


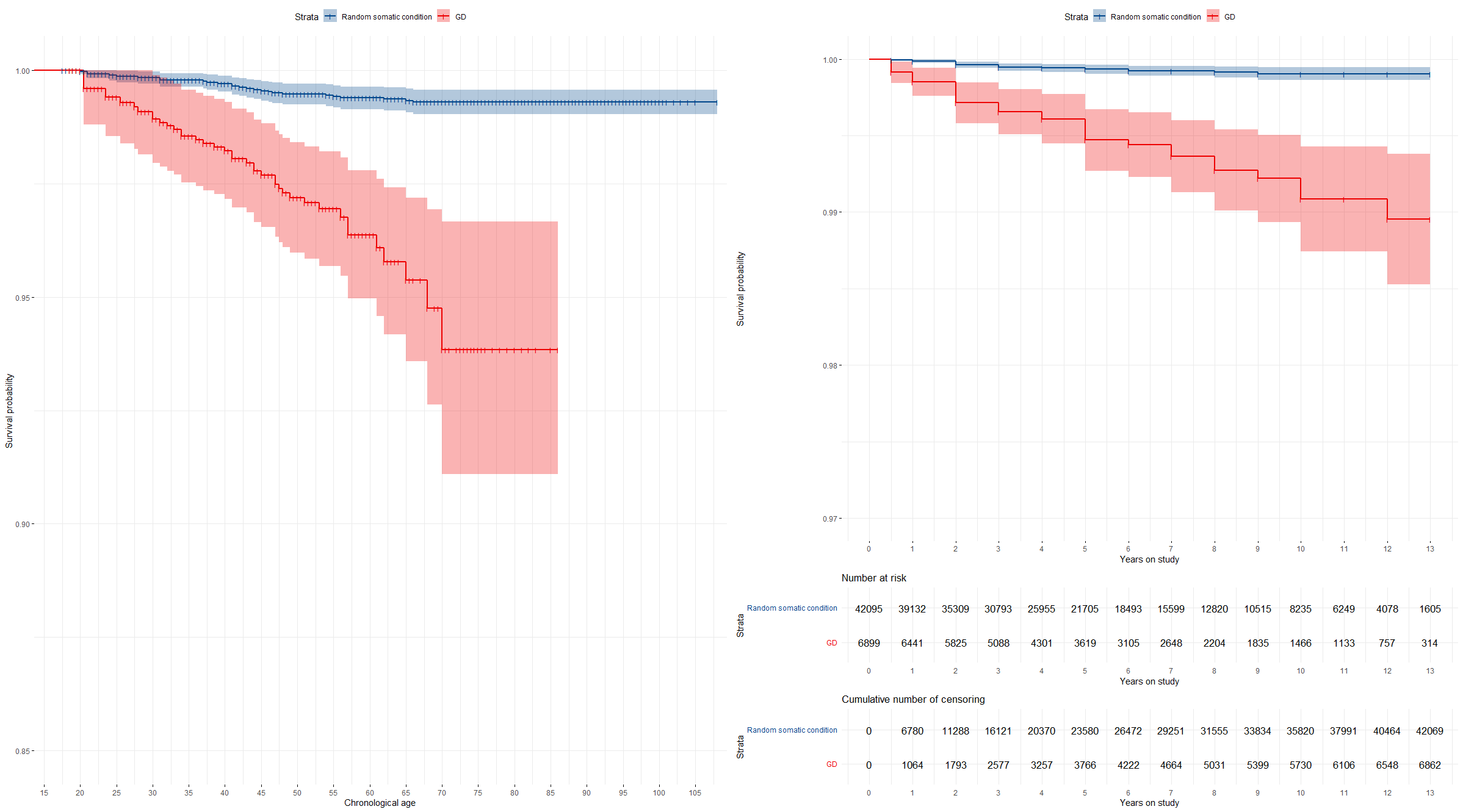


**Supplementary Figure 3**

Kaplan-Meier curves for comparing survival probability of suicide mortality among patients with gambling disorder compared to patients with substance use disorders.


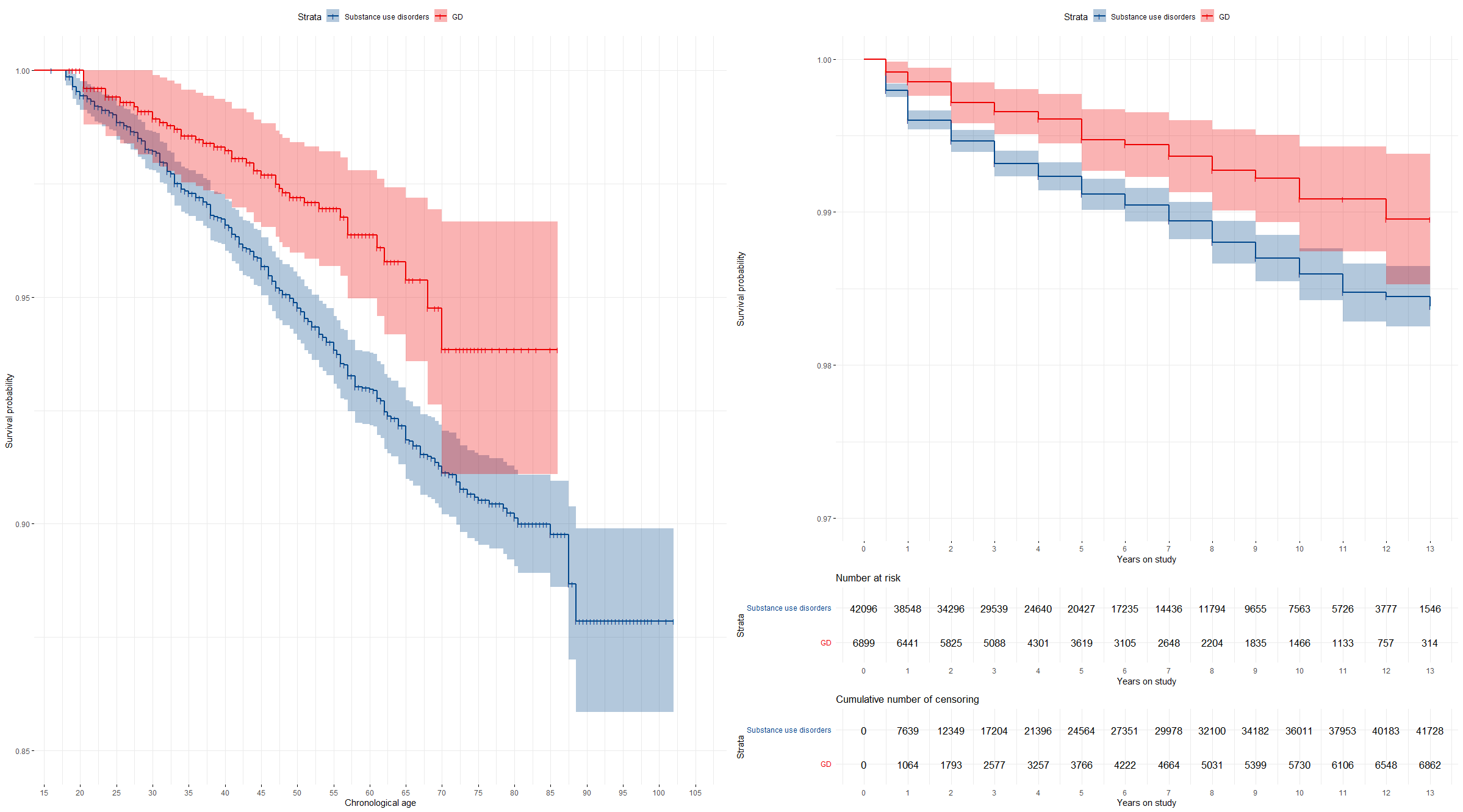


**Supplementary Figure 4**

Kaplan-Meier curves for comparing survival probability of suicide mortality among patients with gambling disorder compared to patients with alcohol dependence syndrome.


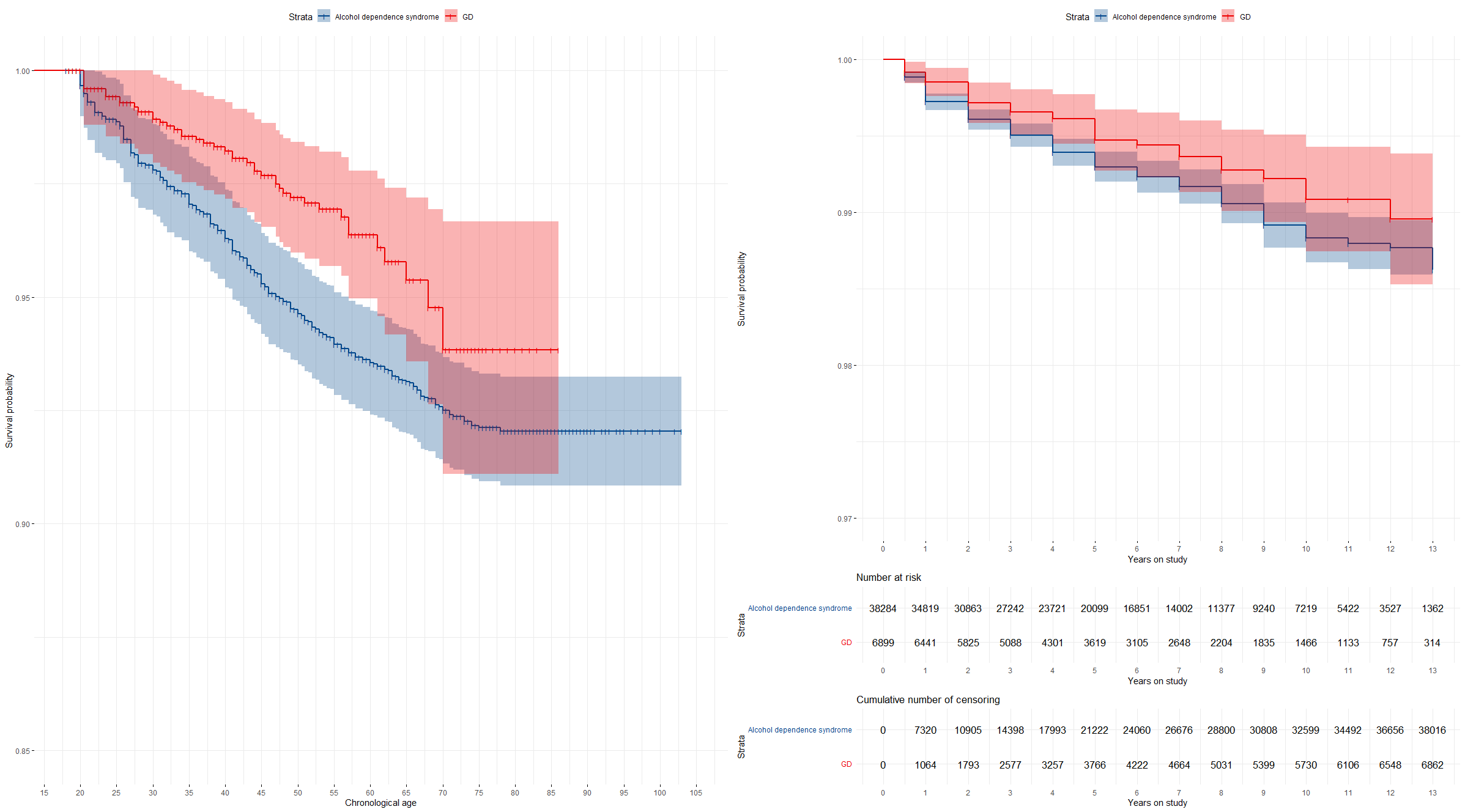


**Supplementary Figure 5**

Kaplan-Meier curves for comparing survival probability of suicide mortality among patients with gambling disorder compared to patients with psychotic disorders.


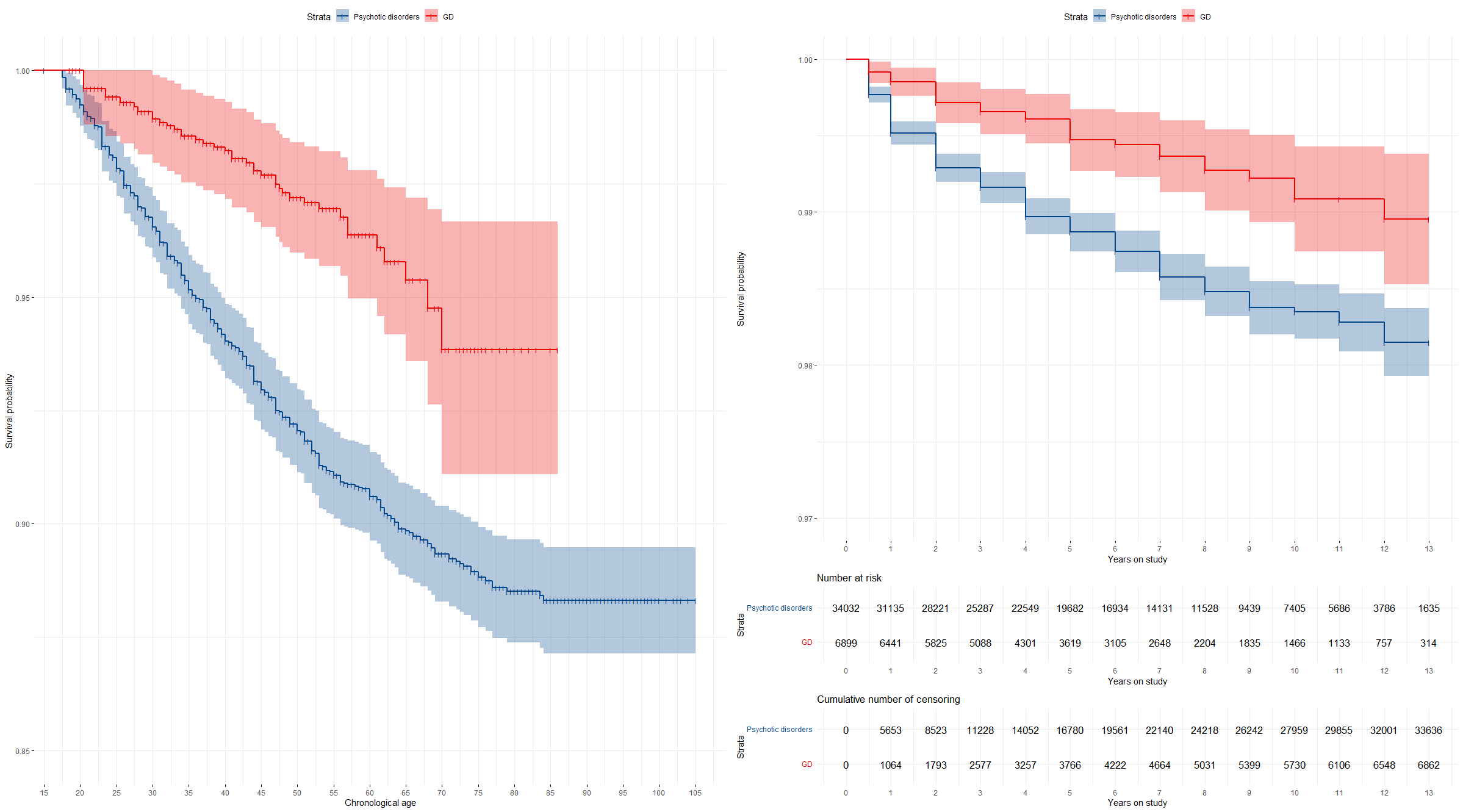


**Supplementary Figure 6**

Kaplan-Meier curves for comparing survival probability of suicide mortality among patients with gambling disorder compared to patients with mood disorders.


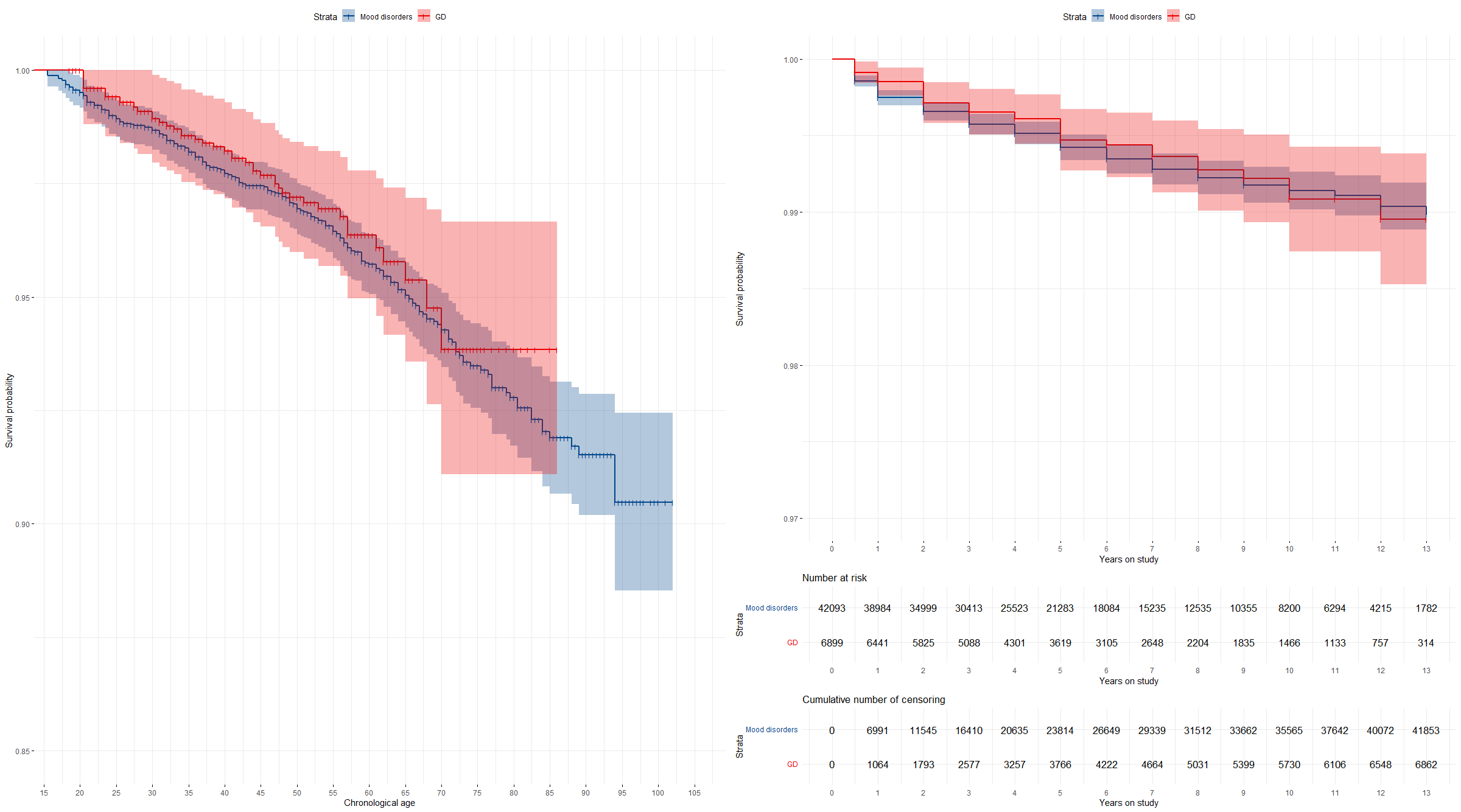


**Supplementary Figure 7**

Kaplan-Meier curves for comparing survival probability of suicide mortality among patients with gambling disorder compared to patients with depression.


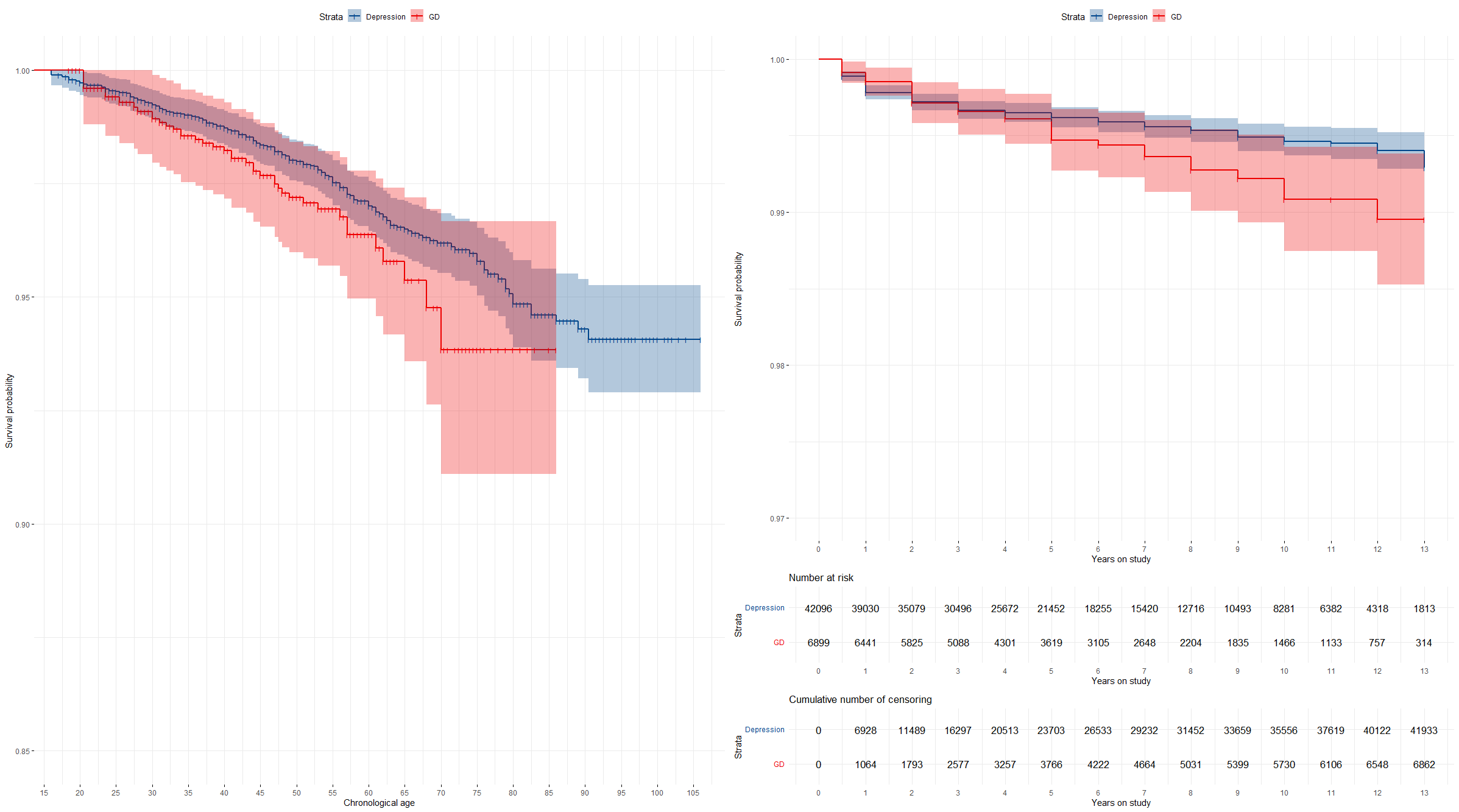


**Supplementary Figure 8**

Kaplan-Meier curves for comparing survival probability of suicide mortality among patients with gambling disorder compared to patients with anxiety disorders.


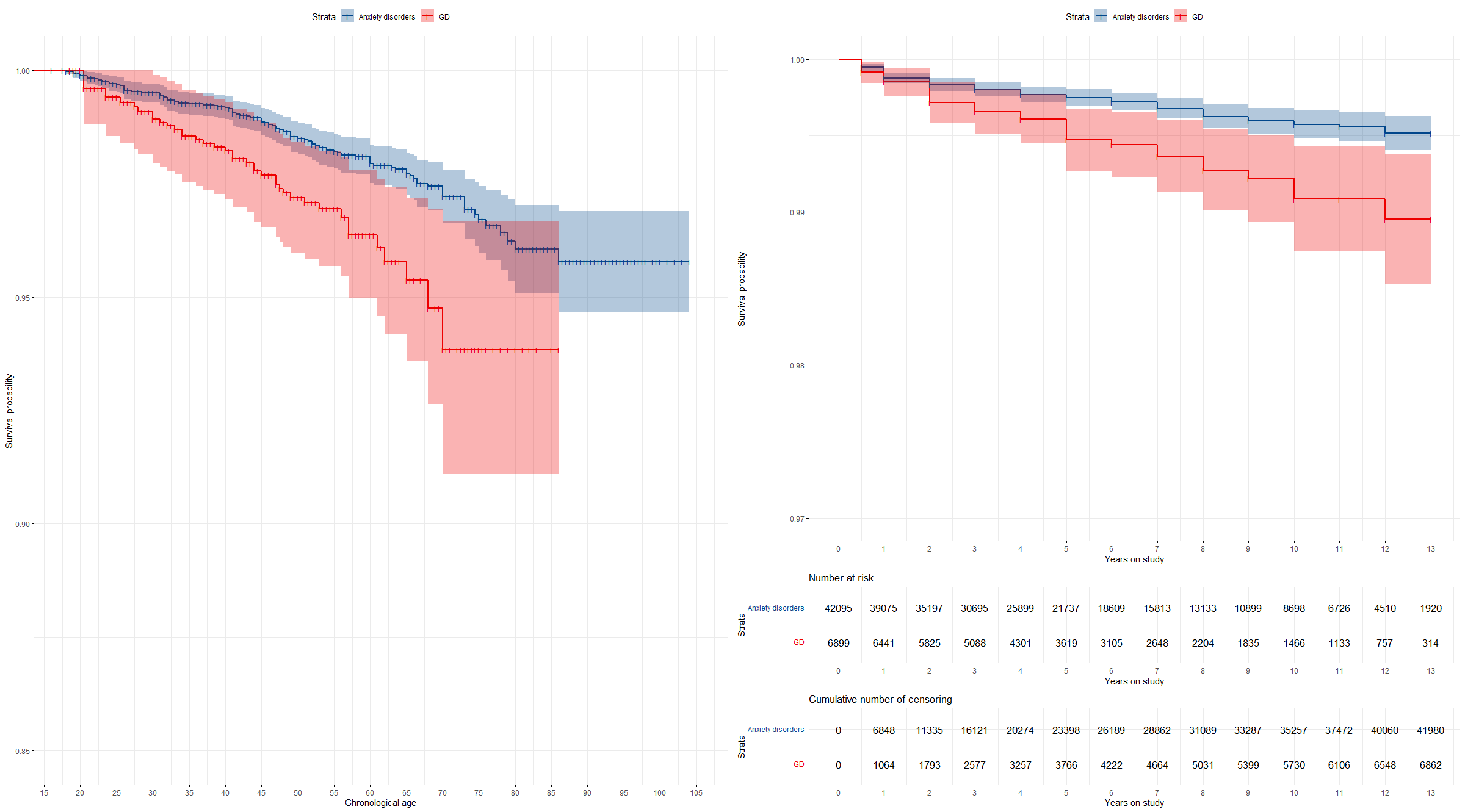


**Supplementary Figure 9**

Kaplan-Meier curves for comparing survival probability of suicide mortality among patients with gambling disorder compared to patients with behavioral syndromes associated with physiological disturbances and physical factors.


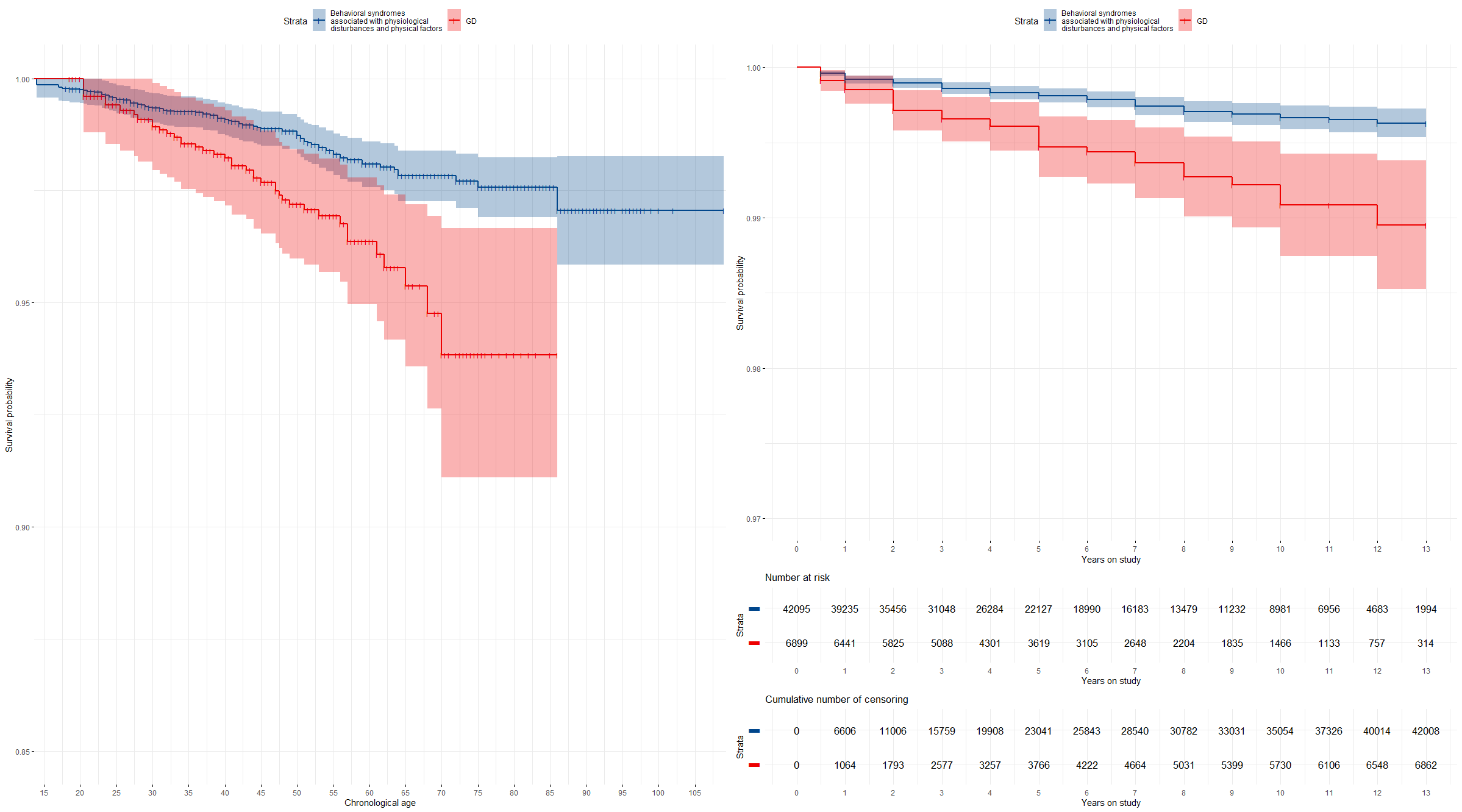


**Supplementary Figure 10**

Kaplan-Meier curves for comparing survival probability of suicide mortality among patients with gambling disorder compared to patients with personality disorders.


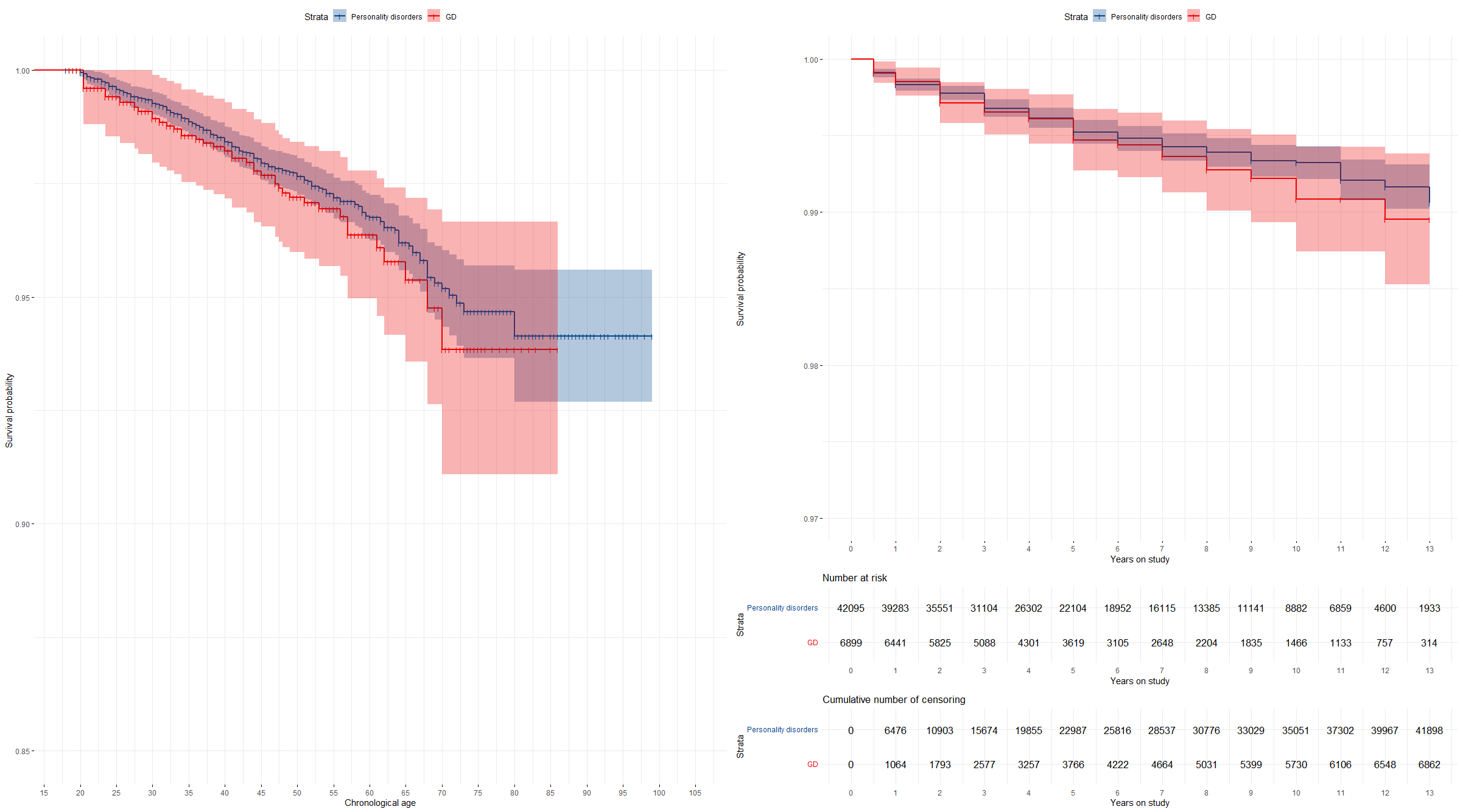


**Supplementary Figure 11**

Kaplan-Meier curves for comparing survival probability of suicide mortality among patients with gambling disorder compared to patients with developmental disorders.


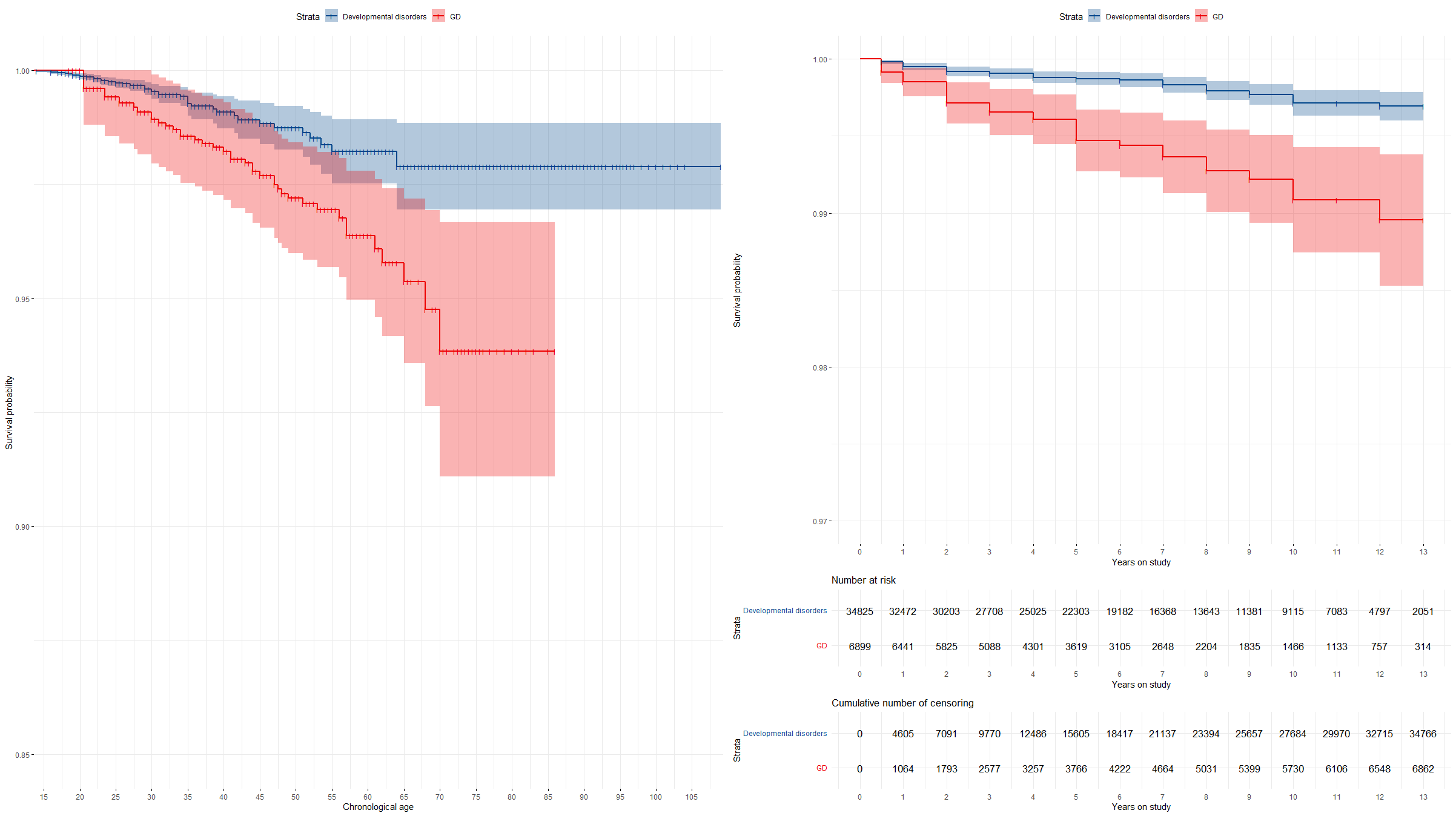


**Supplementary Figure 12**

Kaplan-Meier curves for comparing survival probability of suicide mortality among patients with gambling disorder compared to patients with behavioral and emotional disorders with onset usually occurring in childhood and adolescence.


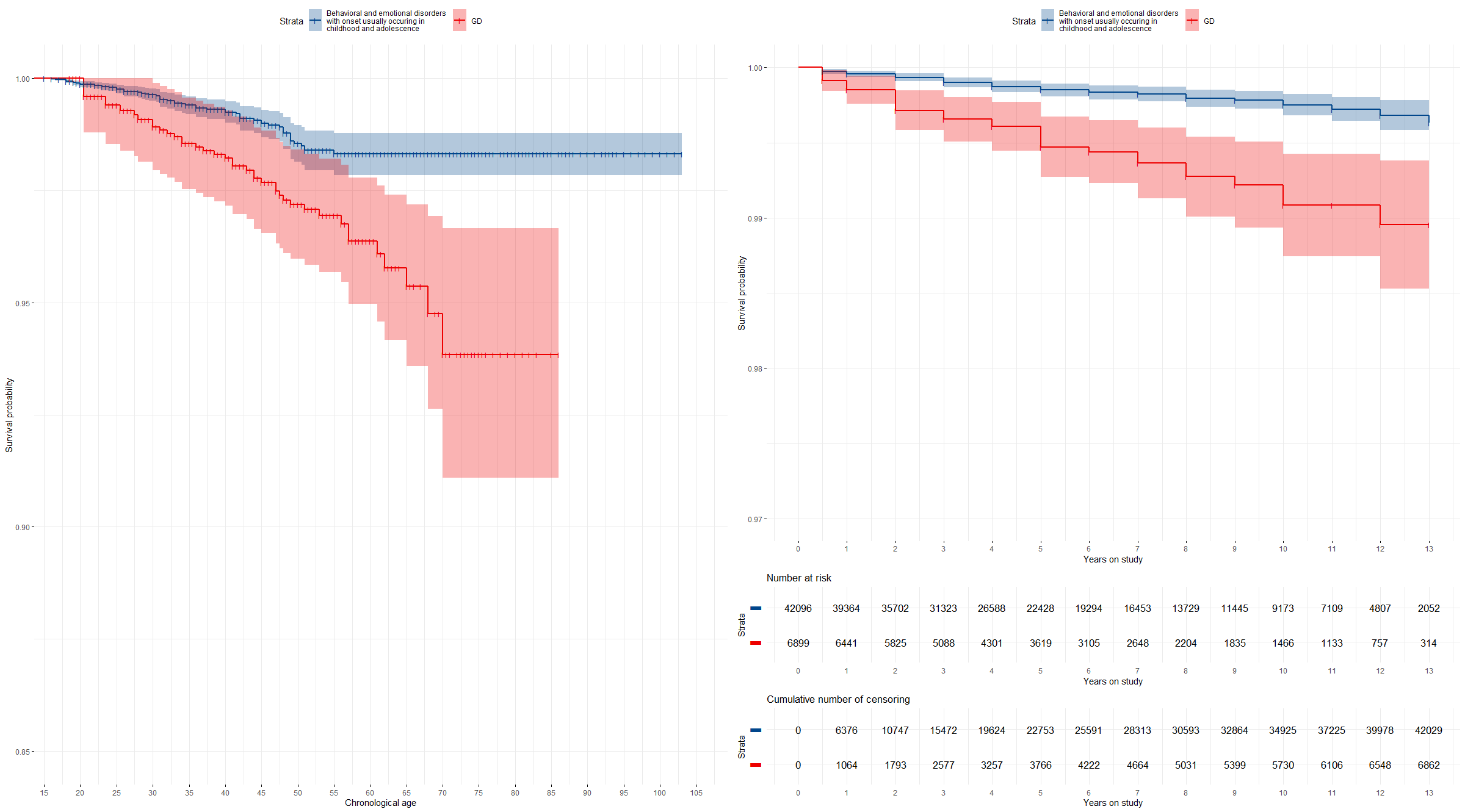


**Appendix D: STROBE Checklist**

STROBE Statement—Checklist of items that should be included in reports of ***cohort studies***

|  | Item No | Recommendation | Location in manuscript |
| --- | --- | --- | --- |
| **Title and abstract** | 1 | (*a*) Indicate the study’s design with a commonly used term in the title or the abstract | Title states cohort study |
|  |  | (*b*) Provide in the abstract an informative and balanced summary of what was done and what was found | Abstract |
| Introduction | | |  |
| Background/rationale | 2 | Explain the scientific background and rationale for the investigation being reported | Background, paragraphs 2-5 |
| Objectives | 3 | State specific objectives, including any prespecified hypotheses | Background, final paragraph |
| Methods | | |  |
| Study design | 4 | Present key elements of study design early in the paper | ‘Study design and data sources’: paragraph 1 |
| Setting | 5 | Describe the setting, locations, and relevant dates, including periods of recruitment, exposure, follow-up, and data collection | ‘Study design and data sources’ |
| Participants | 6 | (*a*) Give the eligibility criteria, and the sources and methods of selection of participants. Describe methods of follow-up | Methods: ‘Study Population and Comparison Groups’ |
|  |  | (*b*) For matched studies, give matching criteria and number of exposed and unexposed | Not applicable |
| Variables | 7 | Clearly define all outcomes, exposures, predictors, potential confounders, and effect modifiers. Give diagnostic criteria, if applicable | ‘Study design and data sources’: second paragraph + ‘Data Analysis’ |
| Data sources/ measurement | 8* | For each variable of interest, give sources of data and details of methods of assessment (measurement). Describe comparability of assessment methods if there is more than one group | ‘Study design and data sources’ |
| Bias | 9 | Describe any efforts to address potential sources of bias | Not applicable |
| Study size | 10 | Explain how the study size was arrived at | ‘Study Population and Comparison Groups’: paragraphs 2 and 4 |
| Quantitative variables | 11 | Explain how quantitative variables were handled in the analyses. If applicable, describe which groupings were chosen and why | ‘Data Analysis’ |
| Statistical methods | 12 | (*a*) Describe all statistical methods, including those used to control for confounding | ‘Data Analysis’ |
|  |  | (*b*) Describe any methods used to examine subgroups and interactions | ‘Data Analysis’: paragraph 2 |
|  |  | (*c*) Explain how missing data were addressed | ‘Data Analysis’ |
|  |  | (*d*) If applicable, explain how loss to follow-up was addressed | Not applicable |
|  |  | (*e*) Describe any sensitivity analyses | ‘Data Analysis’ |
| Results | | |  |
| Participants | 13* | (a) Report numbers of individuals at each stage of study—eg numbers potentially eligible, examined for eligibility, confirmed eligible, included in the study, completing follow-up, and analysed | ‘Study Population and Comparison Groups’: paragraph 2 |
|  |  | (b) Give reasons for non-participation at each stage | ‘Study Population and Comparison Groups’: paragraph 2 |
|  |  | (c) Consider use of a flow diagram | Not applicable |
| Descriptive data | 14* | (a) Give characteristics of study participants (eg demographic, clinical, social) and information on exposures and potential confounders | ‘Descriptive Analyses of Patients with Gambling Disorder’ + Table 1 |
|  |  | (b) Indicate number of participants with missing data for each variable of interest | ‘Data Analysis’: last paragraph |
|  |  | (c) Summarise follow-up time (eg, average and total amount) | ‘Descriptive Analyses of Patients with Gambling Disorder’, + Table 2, Table 3 |
| Outcome data | 15* | Report numbers of outcome events or summary measures over time | ‘Descriptive Analyses of Patients with Gambling Disorder’ + Table 2, Table 3 |
| Main results | 16 | (*a*) Give unadjusted estimates and, if applicable, confounder-adjusted estimates and their precision (eg, 95% confidence interval). Make clear which confounders were adjusted for and why they were included | ‘Risk of Suicide Mortality Compared to the General Population’ + ‘Risk of Suicide Mortality Compared to Other Patient Groups’ + Table 2, Table 3 |
|  |  | (*b*) Report category boundaries when continuous variables were categorized | Table 2 |
|  |  | (*c*) If relevant, consider translating estimates of relative risk into absolute risk for a meaningful time period | Not applicable |
| Other analyses | 17 | Report other analyses done—eg analyses of subgroups and interactions, and sensitivity analyses | ‘Risk of Suicide Mortality Compared to the General Population’ + ‘Risk of Suicide Mortality Compared to Other Patient Groups’, + Supplementary Table 1, Supplementary Table 2 and ‘Study Population and Comparison Groups’ |
| Discussion | | |  |
| Key results | 18 | Summarise key results with reference to study objectives | Discussion: paragraph 1 |
| Limitations | 19 | Discuss limitations of the study, taking into account sources of potential bias or imprecision. Discuss both direction and magnitude of any potential bias | Discussion: paragraphs 4 to 8 |
| Interpretation | 20 | Give a cautious overall interpretation of results considering objectives, limitations, multiplicity of analyses, results from similar studies, and other relevant evidence | Discussion: final paragraph |
| Generalisability | 21 | Discuss the generalisability (external validity) of the study results | Discussion: paragraphs 4-6 |
| Other information | | |  |
| Funding | 22 | Give the source of funding and the role of the funders for the present study and, if applicable, for the original study on which the present article is based | ‘Role of funding source’ + Author statements |

*Give information separately for exposed and unexposed groups.

**Note:** An Explanation and Elaboration article discusses each checklist item and gives methodological background and published examples of transparent reporting. The STROBE checklist is best used in conjunction with this article (freely available on the Web sites of PLoS Medicine at http://www.plosmedicine.org/, Annals of Internal Medicine at http://www.annals.org/, and Epidemiology at http://www.epidem.com/). Information on the STROBE Initiative is available at http://www.strobe-statement.org.
